# Advance rent mobilisation strategies of graduate renters in Ghana: a submarket of the private rental housing market

**DOI:** 10.1007/s10901-021-09926-w

**Published:** 2021-12-30

**Authors:** Lewis Abedi Asante, Richmond Juvenile Ehwi, Emmanuel Kofi Gavu

**Affiliations:** 1 Department of Estate Management, Kumasi Technical University, Kumasi, Ghana; 2 Cambridge Centre for Housing and Planning Research, Department of Land Economy, University of Cambridge, Cambridge, UK; 3 Department of Land Economy, Kwame Nkrumah University of Science and Technology, Kumasi, Ghana

**Keywords:** Advance rent, Private rental housing, Graduate renters, Mobilization strategies, Social capital, Ghana

## Abstract

The practice of advance rent, where landlords ask renters to pay a lump-sum rent covering 2 or more years, is gaining scholarly and political attention in Africa. Nevertheless, there is limited empirical research investigating how renters mobilize funds to meet this financial commitment. Existing literature suggests that renters, irrespective of their educational level, face difficulties in paying advance rent, hence compelling them to rely mainly on their bonding (family and friends) and bridging (employers and financial institutions) social capital to pay advance rent. Drawing on rational choice and social capital theories coupled with data from a novel (graduate) sub-market of Ghana’s rental housing market, this article finds that personal savings remain the most rational current and future source of funding options graduate renters draw upon to pay advance rent, albeit some still drawing on their social capital. The findings demonstrate that graduate renters do not use bonding social capital in their future mobilization strategies after they have drawn on the same in previous years, although they continue to rely on their bridging social capital and other strategies to mobilize funds for advance rent. The study suggests the need to rethink rational choice and social capital theories to incorporate inter-temporal dynamics among different social groups and to traverse the current binary conception of the rental housing market in Ghana to consider different sub-markets and how they respond to existing challenges in the housing sector.

## Introduction

The importance of rental housing as a viable tenure option to societies around the globe remains undoubted. It provides accommodation at a relatively cheaper cost than homeownership; houses all income groups in urban populations; and is generally accepted as a fundamental determinant of overall quality of life (Andreasen et al., 2020; Baiden et al., 2011; Ehwi et al., 2020; Obeng-Odoom, 2011). Nevertheless, rental housing in cities in Sub-Saharan Africa (SSA) is bedevilled with several challenges. The majority of urban residents live in deteriorated and overcrowded rental dwellings without adequate access to basic amenities such as sanitation, water and waste disposal systems (Addo, 2016; Luginaah et al., 2010; Obeng-Odoom & Amedzro, 2011). Most rental accommodations are also rented with few or no furnishings, compelling first-time and regular renters to bear a huge cost of furnishing during their tenancy period (Ehwi et al., 2020). Publicly available information on rental housing is limited, and this forces renters to resort to personal search, and information from friends, family members and estate agents to find accommodation (Adu-Gyamfi et al., 2020; Gavu et al., 2019). This notwithstanding, the effective demand for rental housing in African cities continues to exceed supply, resulting in a situation where urban residents have to compete for the available few rental accommodations at high rents (World Bank, 2014). Notably, we contend that the crux of the rental housing problem in many African cities is not only about the inadequate basic amenities, the lack of furnishing, the difficulty in finding information on vacant houses or high rents, but also the long periods over which renters have to pay their rent upfront.

Advance rent (hereafter AR) is defined as a capital or lump-sum payment of rents at the start of a tenancy (Asante, Gavu, Quansah, & Osei Tutu, 2018). It is obtained by multiplying the monthly rent by the tenancy period. The practice of AR is prevalent across several countries and cities in SSA, albeit with some variations in the tenancy period (Africanews, 2018; Arku et al., 2012; Cadstedt, 2006; Cain, 2017; Cartus, 2017; Olawande et al., 2012). In Senegal for instance, renters pay 3 months of AR to landlords, although the tenancy period ranges from 12 to 36 months (Cartus, 2017). In Tanzania, Cameroon and Angola, renters pay between 6 and 12 months of AR (Africanews, 2018; Cadstedt, 2006; Cain, 2017). Studies by Arku et al. (2012), Olawande et al. (2012) and UN Habitat (2011) have shown that the most extreme and exploitative form of the practice of AR is found in Ghana and Nigeria – where renters pay between 24 and 60 months of AR, with an increment of between 55 and 75% at the end of each contract period. These studies further show that the period of AR charged by landlords contravenes the rent legislation in both Ghana and Nigeria (Kufuor, 2018). In Ghana, for example, Section 25(5) of the Rent Act, 1963 (As amended) permits landlords to charge AR of not more than 1 month for shorter tenancies and 6 months for longer tenancies.

Despite the prevalence of AR payment in the rental housing markets of several African cities, there has been little empirical inquiry into how renters mobilise funds to pay this hefty financial commitment. Instead, the existing literature has tended to focus on issues such as the psychological impacts of the AR on renters (Luginaah et al., 2010), the impact of the AR payment on renters’ lived experiences (Arku et al., 2012), the financial burdens imposed on renters by paying the AR and furnishing their rental dwellings simultaneously (Ehwi et al., 2020) and how landlords use their relative power to extract AR (Owusu-Ansah et al., 2018). The few studies that have reflected on how renters pay the AR (Arku et al., 2012; Owusu-Ansah et al., 2018) suggest that renters, irrespective of their educational level, face difficulties in paying advance rent, hence compelling them to rely mainly on their family members, friends, employers and banks to raise funds for the payment of AR. Notably, these studies did not have the AR payment as their primary focus and hence are unable to yield an in-depth and conceptually grounded analysis of both the logic behind the mobilisation strategies adopted and the relative importance of different funding sources renters draw upon to pay the AR. Also, the analysis of sources of funds for the AR payment and strategies discussed in these studies do not account for the vicious cyclical nature of the AR payment and hence, the need for temporal analysis of both the mobilization strategies and the source of funding used.

This research seeks to fill this gap by posing two research questions: (1) What strategies do graduate renters currently adopt to mobilize funds to pay their AR? (2) Are there changes in both the strategies graduate renters use to mobilise funds and the relative importance of the sources of funds they draw upon to pay their AR? In answering these questions, we draw both theoretical and conceptual insights from rational choice and social capital theory (See Sect. 3 for elaboration) to explore both the strategies mobilised and the logic behind the sources of funding drawn upon. We also use a novel cohort of renters – graduate renters – to explore these questions. By graduate renters, we mean households where the household head (or household member who pays part of the rent) has completed tertiary education (See more on the selection of this cohort in Sect. 4). We will learn that graduate renters rely on mainly personal savings as the most rational current and future source of funds to pay AR. Nevertheless, where personal savings are inadequate, they fall on their social capital. Using this cohort of renters is significant because it coincides with policy propositions by the two mainstream political parties in Ghana – the incumbent New Patriotic Party (NPP) and the opposition National Democratic Congress (NDC) – to assist renters with regular incomes (majority of whom are likely to be people with tertiary education) to make an upfront payment of the AR, while they repay monthly (Joy News, 2020; National Democratic Congress, 2020; New Patriotic Party, 2020). Thus, insights from this study can help policymakers better understand the sources of funding graduate tenants draw upon to pay the AR, leading to better targeting of the proposed rental housing policy.

The rest of the paper is organized as follows. Section two illuminates Ghana’s rental housing market and the emergence of the AR practice and how renters have typically mobilised funds to pay this financial commitment. In Sect. 3, we reflect on both rational choice and social capital theories to conceptualize AR mobilization strategies, paying attention to its potential vicious cyclical nature. Section 4 describes the study area and outlines the research methods. The findings of the research are presented in Section five while Section six discusses how our results concur with and differ from existing scholarship on the rental housing market in Ghana generally. Section seven concludes by reflecting on implications of the findings for private rental housing, social capital theory and research methods during crisis times.

## Rental housing and advance rent in Ghana

### Overview of rental housing in Ghana

In 2010, Ghana became one of the few countries in sub-Saharan Africa (SSA) with more than half of its population living in urban areas (Asante, 2020; Ghana Statistical Service, 2014a). During the last decade, Ghana’s population has increased from about 24.7 million in 2010 to 30.8 million in 2021, and more than one-third of the population live in the Greater Accra or Ashanti Region (Ghana Statistical Service, 2021). The heavy concentration of people in Ghana’s biggest cities based on provisional results from the 2021 Population and Housing Census also suggests that Ghana’s urban population is set to also increase from the 56.7% recorded in 2010 (Ghana Statistical Service, 2012). The census report also reveals that there are now 8.6 million fully completed structures in Ghana and residential structures constitute 57% of the stock which is equivalent to 4.9 million structures (Ghana Statistical Service, 2021). This growing population will inevitably result in widening the housing deficit which anecdotal evidence suggests now stands at 2 million (Kufuor, 2018).

Housing delivery in Ghana has predominantly been delivered by individual households and families (Acheampong & Anokye, 2015; Tipple & Korboe, 1998). Attempts by the state to provide affordable housing mostly targeted top civil servants and largely focused on homeownership (Boamah, 2014; Konadu-agyemang, 1990). The neoliberal economic policies adopted in the 1980s also ushered in private-sector driven housing supply which predominantly targeted the affluent households and promoted homeownership over other forms of tenures (Arku, 2009). Consequently, the private informal sector now provides more than 80% of the entire housing stock in Ghana (Ehwi et al., 2020; UN Habitat, 2011) and caters to the needs of most low-income households (Andreasen et al., 2020; Yankson, 2011).

The rental housing sector in Ghana has been thought of as bifurcating into public rental housing on one hand, and the private formal and informal rental housing on the other hand (Arku et al., 2012). The former comprises state-funded estates targeted at civil servants and accounting for about 3% of the housing stock (Ehwi et al., 2020). The private formal rental housing comprises villas, apartments and (semi) detached dwellings developed by private real estate developers and let to affluent and rising middle-class households (Ghana Statistical Service, 2014b; Tipple & Willis, 1992). The private informal sector comprises dwellings built by private landlords and families who build incrementally, often outside the formal planning system (Andreasen et al., 2020; Arku et al., 2012). They target households of all income groups, accounting for about 80% of the rental housing stock in Ghana (Ehwi et al., 2020; UN-Habitat, 2011). Owing to the dominant share of this market vis-à-vis the growing housing deficit and poor regulation of the rental housing sector, landlords arbitrarily demand AR of up to 2-years despite lacking basis in the current Rent Act, 1963.

### The genesis of the advance rent system and advance rent mobilization strategies

Since the 1960 s, renting in Ghana has been regulated by the Rent Act, 1963 (Act 220) (As amended). Initially, the law did not prohibit landlords from charging long periods of AR. During the turbulent political period in Ghana (1982-83), landlords took advantage of the shortcomings of the law and the shortage of housing across major cities to increase rents arbitrarily, thus pricing many low- and middle-income households into ‘worse’ forms of housing. At the time, the government responded by enacting the (i) Rent Control Law, PNDC Law 5 (ii) the Compulsory Letting of Unoccupied Rooms and Houses Law, PNDC Law 7 and (iii) the Rent Tax Law 1984 PNDC Law 82 to control the operations of the rental housing market (Ninsin, 1989). As noted by Kufuor (2018), landlords were compelled to reduce rents to levels deemed to be within the means of low-income renters, and in some cases, state officials forcibly took possession of unoccupied dwellings and let them to prospective tenants. The activism of landlords’ associations in cities across Ghana led to the passage of the Rent Control Law, 1986, PNDC Law 138, which abolished the protection enjoyed by tenants under the old laws (Ninsin, 1989). However, Section 19(2) of the PNDC Law 138 amended Section 25(5) of Act 220 by making it an offence for any landlord in Ghana to demand more than 1 month AR for shorter tenancies and 6 months for longer tenancies.

Several studies have shown that landlords in Ghana flout this provision of Act 220 and PNDC Law 138 with impunity (Asante et al., 2018; Gavu & Owusu-Ansah, 2019). It is believed that the dominance of landlords in the supply of housing in Ghana and the inefficiency of the state apparatus clothed with the power to protect tenants account for the total disregard of the laws (Kufuor, 2018). Until recently, successive governments have made little effort to address the problem of AR. The non-enforcement of provisions in the Rent Act has brought untold financial hardship on renters who are compelled to pay the 2-year AR.

Existing studies have drawn attention to the difficulties renters face in paying the 2-year AR. For example, both Owusu-Ansah et al. (2018) and Arku et al. (2012) have shown that, while a few renters in Accra found no problem with the 2-year AR because it froze monthly rent payment for 2 years, the majority complained about the tremendous financial difficulties associated with payment of AR. Ehwi et al. (2020) capture this difficulty succinctly by arguing that first-time and regular renters in Dansoman, a suburb of Accra, have to save over 9 months and 7 months of their net income respectively to pay the 2 years’ AR. Furthermore, Asante & Ehwi (2020) have also demonstrated that the housing transformations taking place in rental accommodations in Kumasi are doubling monthly rents and increasing the AR beyond the financial capability of low-income households. They further argue that *‘both the poor and rich are made financially worse off due to the practice of AR’* (p. 20). Consequently, Luginaah et al. (2010) reported that the numerous housing problems, including the payment of AR, confronting renters predispose them to psychological distress and diminishing ontological security.

In terms of how renters mobilised funds to pay the AR, there is consensus within the existing literature that renters generally solicit financial assistance from their relatives, friends, and employers. For example, in their study on renters’ experiences, and landlord-tenant relations in Adabraka, Accra, Arku et al. (2012) reported the circumstance where tenants whose salaries could only pay half of the AR had to raise the other half from their relatives, especially those living abroad. Similarly, the study by Owusu-Ansah et al. (2018) on the relative power of landlords and tenants to shape the rental housing market, found that 60% of the 344 renters they sampled from Accra and Tema relied on friends and family members to raise the AR while the remaining 40% had to take moderate interest loans from their employers. In a recent study by Asante & Ehwi (2020), they indicated that some renters resort to bank loans to pay their AR. As insightful as these funding sources reported seem, their lack of theoretical grounding makes it difficult to interrogate the logic behind the different strategies that renters use, the sources of funds they draw upon, and how these strategies change over time as the AR assumes a cyclical nature. Following these shortcomings, we present our theoretical and conceptual framework to explore the logic behind the different AR mobilisation strategies and sources of funds renters draw upon to make this payment.

## Theoretical underpinning of the research

### Theories of rational choice and social capital

In exploring the strategies renters draw upon to mobilize funds to pay the AR, we propose a conceptual framework that draws insights from both rational choice and social capital theories. Rational choice is a well-founded theory widely applied in social science disciplines, predominantly in neoclassical economics, to explain the decision-making process of the ‘economic man’ (Green & Shapiro, 2014; Simon, 1952). For this economic man, the theory makes assumptions about the motives for engaging in economic activities, his cognitive and predictive abilities, the set of choices he faces and the outcomes he desires (Simon, 1952). The economic man is first assumed to be egoistic and driven by the quest to maximize his utility while minimizing costs in any economic activity. He is also presumed to possess adequate information about the choices he faces in his decision-making process, the behaviour of other actors he engages with, and an ability to calculate the probabilities of success and failure associated with his options. While rational choice has for a long time provided a foundational theory for most human actions, it is criticized for, among other things, overstating the cognitive and calculative abilities of the rational man and downplaying the transaction costs associated with each decision alternative, which can prove abortive without the mediation of institutions or other forms of social arrangements (Alasuutari, 2015; Coarse, 1960).

We aim to overcome the above limitation of rational choice by drawing on social capital theory. Putting it plainly, de Souza Briggs (1998) observed that social capital is ‘what we draw on when we get others, whether acquaintances, friends, or kin, to help us solve problems, seize opportunities, and accomplish other aims that matter to us’ (p.178). Putnam (2000) points out that social capital is reflected in the network of relations we build and the norms of reciprocity that underlie interpersonal, person-to-group and intergroup relations. This network of relations and norms of reciprocity have conceptually been viewed in terms of whether it takes place within a small, homogenous, dense and strong network (bonding social capital), a large, unbounded, diffused and weak networks (bridging capital) (Putnam, 2000), or a specific hierarchical power structures (linking capital) (Agger & Jensen, 2015). It has been argued that bonding capital is common among groups who have a lot in common as in the case of families, friends and members of a club (Putnam, 2000). Bonding capital is therefore inward-looking and fosters exclusivity and loyalty to group members (Putnam, 2000). Thus, bonding social capital is useful for ‘getting by’ (de Souza Briggs, 1998). Relationships within bonding capital are mostly informal and can often be counted upon (Agger & Jensen, 2015). Bridging capital is more outward-looking, seeking to connect one person with the assets, resources and information of others outside his network. It is common among acquaintances and relationships are mostly formal (Agger & Jensen, 2015), although they can also be informal. Linking capital affords people the opportunity to engage with political elites and power holders such as local authorities to address local problems (Agger & Jensen, 2015). Relationships are often formal and structured with clearly defined mandates and expectations.

Despite this conceptual distinction between these three forms of capital, they do not play out so distinctly in practice as it is possible to combine two or all three forms of social capital, albeit in varying proportions to realize an expected goal (Putnam, 2000). In this study, we adopt bonding and bridging social capital, where the former relates to support from family members and friends while the latter connotes support from employers and banks.

### Conceptualizing advance rent mobilization strategies

In applying both rational choice and social capital theories to conceptualize the strategies renters use to mobilize funds to pay their AR, we first posit that social capital is not mutually exclusive from rational choice (Lin, 2004). This is because the decision to use any form of social capital is first rational, meaning one is putting their needs above others by exploring how to maximize those needs at the barest minimum cost. According to Lin (2004), individuals are motivated to invest in social networks or relations because they expect a return in the form of social capital. However, where we depart from the axioms of rational choice, is the perfect information regarding alternative courses of action available to the rational man. We rather argue that, at any given point, one would not know all the alternative courses of action required to achieve a stated goal, as some of the information needed would be embedded in existing social networks which must be tapped into and used to complement existing knowledge. In Fig. 1, we argue that renters’ first strategic option in mobilizing funds to pay their AR is to use their savings. This is because, aside, the predictability and accessibility of this alternative, it could hurt one’s pride or even be construed as a sign of irresponsibility when one depends entirely on the benevolence of others to meet an obvious basic need such as housing. Thus, renters are in effect supposed to personally contribute by showing commitment to how much they have saved towards AR payment before seeking third party help and support. This implies that the probability of obtaining funds from one’s savings to pay AR is or approximates to one.

Unfortunately, high levels of unemployment or under-employment and low wages in Ghana and indeed in many SSA countries make it difficult for people to rely solely on their savings to fund AR payment and other financial commitments associated with housing (Irwin, Mader, & Flynn, 2018). In Ghana, for instance, incomes are generally low among all sections of society, as middle-income earners take home an average of $264.17 per month (World Bank, 2014). The Ghana Living Standard Survey (GLSS) 7 reports that the main reason why most Ghanaians are unable to save is that they do not earn enough income (Government of Ghana, 2019). Owing to these exigencies, people will be compelled to draw on their social capital to be able to complement their savings or in some rare cases, pay the full AR through their social networks. We postulate in this respect that renters would first draw on their bonding social capital, namely financial assistance from family members, friends, work colleagues, religious or faith-based groups to raise the additional funds to complement their savings to pay AR (Arku et al., 2012; Luginaah et al., 2010; Owusu-Ansah et al., 2018). Here, we classify religious or faith-based groups as bonding social capital because of the strong ties that bind people. Faith-based groups in Ghana are not premised on legalities but on social values like empathy, fellow-feeling, selflessness all of which affords easy access to financial aid, often without an obligation to repay. Drawing on one’s bonding social capital will remain so for both the employed and unemployed because accessing funds within one’s bonding capital does not require going through formal processes or meeting specific requirements such as ability to repay. This means that there is a higher likelihood of success associated with bonding social capital. However, it is worth mentioning that the likelihood of funding success using bonding social capital will be lower than using one’s savings (Success rate = <1).

In the case of renters who are employed, they would have the third option of using their bridging capital by seeking financial assistance from their employers or financial institutions. We classify financial assistance from employers and financial institutions as bridging capital because there are often legally enforceable contractual obligations that regulate how funding is accessed from these sources. This option has been highlighted in previous research (See Asante & Ehwi, 2020; Owusu-Ansah et al., 2018). While employers don’t need to support their employees with the payment of their AR, we contend that it is often in their business interest to partly or fully fund employees’ AR. Financial institutions likewise are motivated by profits and would enact specific requirements to ensure that they can recoup any financial assistance extended to working renters. Personal loans from financial institutions in Ghana are characterized by high interest rates ranging between 28 and 30% per annum (Asante & Ehwi, 2020; Ayensu, Gbemu, Kuma, & Appiah, 2016). Owing to such formal processes, specific credit requirements and high interest rates, we posit that the probability of renters obtaining financial assistance from these sources may be less than or equals to the probability of success in using one’s bonding social capital.

In this paper, we make a conceptual contribution to the social capital literature by introducing temporal analysis. We do this by exploring changes in both the ordering and proportion of the different forms of social capital renters drawn upon after they have previously used these forms of social capital. We argue, based on our knowledge of the cultural context in Ghana that, the likelihood of renters drawing again on their bonding social capital to fund their AR at a later date will weaken owing to the social stigma associated with always going back to one’s kith and kin for financial help, especially when one is married and/or working. This statement holds if the bonding capital renters access is for free and that they do not repay the loan from relatives, friends and close associates. If the monies are repaid, however, it may instead build confidence in the associates and make the bonding capital a reliable source to fall on always. Bridging social capital, on the other hand, is likely to remain the same or increase in the frequency of use primarily because the contractual nature of the transaction obviates social stigmas. Also, regarding the importance of using savings in the future, we assume that the majority of renters would draw on their savings or explore other avenues to increase their savings to pay for their AR rather than drawing on their social capital. This means that, in the future, renters will cut down or avoid using bonding social capital to help fund the AR payment. This would be less so for bridging capital which is likely to increase in the frequency of use, especially for those still facing difficulties in paying their AR. Consequently, we posit that renters are always navigating between different rational alternatives at their disposal and prioritizing the adoption of one alternative over the other. Our conceptual framework is presented in Fig. 1 below and these postulations are explored later in this paper.

Finally, it is worth making a conceptual point about the relationship between strategies and the sources of funds renters adopt to pay the AR. From a rational choice standpoint, strategies are intentioned plans informed by subjective assessments of the benefits and risks associated with any choice set. In the context of AR payment, the purpose of a strategy is to reduce or eliminate the difficulties associated with AR payment. Following this, the sources of funds drawn upon become the realization of the strategy put in place.

**Figure:**
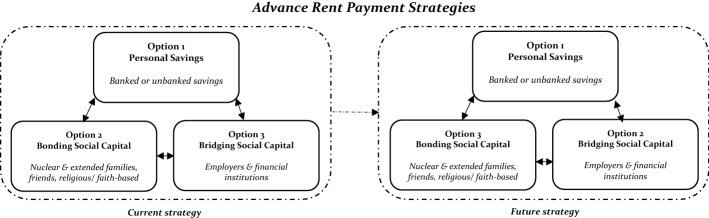
Fig. 1 A conceptual framework of the current and future strategies renters use in mobilizing funds to pay advance rent. *Source*: Authors’ construct. (*Note 1* Solid line rectangles represent different strategies for mobilizing funds to pay the AR while the two broken line rectangles represent different time periods with the current period on the left and future period on the right. *Note 2* Double-headed arrow show potential relationship between the different strategies for mobilizing funds for the AR payment. *Note 3* Single-headed arrow shows the direction of time travel (i.e., from the current to future))

## Research methods

This research was conducted during the peak of the novel Coronavirus (May and July 2020). This meant that it was not possible to adopt in-person data collection approaches having regard to the health and safety protocols in place at the time. The study thus adopted a web-based survey as the most pragmatic approach to gathering data given its advantages such as faster data collection, elimination of the need to manually input data into statistical software for analysis, cost-savings on printing and the ability to have the survey completed by multiple people across different geographies simultaneously (Bhutta, 2012). Despite this, by adopting a web-based survey, the study also faced limitations such as not having a random sample, potentially under-representing some demographic groups such as older people, the uneducated, and those who may have issues with internet privacy (Bhutta, 2012; Wiersma, 2013). However, given that this study is exploratory and not intended to generalise our findings across Ghana, but to share new insights, using web-based surveys served the purpose.

The Qualtrics web-based survey was used to create a questionnaire that sought to gather a range of information, including; renters’ socio-demographic characteristics, their housing circumstances, the source of funds used to pay the AR, perceptions about the difficulties associated with paying the AR, reasons why some residents find the AR payment difficult and the strategies that those who find the AR payment difficult have adopted to deal with the difficulties faced. The survey questions comprised both closed and open-ended questions. The open-ended questions gathered information about the reasons why renters find the AR payment difficult and the strategies they have adopted to avoid future payment difficulties. A unique URL was generated which when followed led respondents to complete the online survey.

Following Bhutta (2012), the chain-referral method was adopted to recruit research participants. This approach entailed each author first forwarding the unique URL to renters within their social networks across all the sixteen regions in Ghana. These contacts were implored to share the URL with other renters in their social networks, leading to a remote snowballing approach. To maximise the survey’s visibility, all authors also pinned the survey to their Twitter handles and also shared the survey link on social media platforms such as WhatsApp, Facebook, Twitter, Instagram, LinkedIn and Telegram. To qualify to take the survey, one needed to confirm that: (1) they lived in privately rented accommodation and (2) were living in Ghana at the time of the survey. Respondents had to respond in the affirmative to both questions to qualify for the survey.

The survey remained open from 23 May to 15 July 2020. At the close of the survey, a total of 651 people had opened the survey. Of this number, 135 people representing 20.74% did not meet the eligibility criteria and were thus made to exit from the survey. Of the remaining 516 who participated in the study, 140 people representing 27.13% did not fully complete all sections of the survey and hence their responses were excluded in the analysis, leaving us with 376 complete valid responses, representing a 72.87% response rate.

**Fig. 2:**
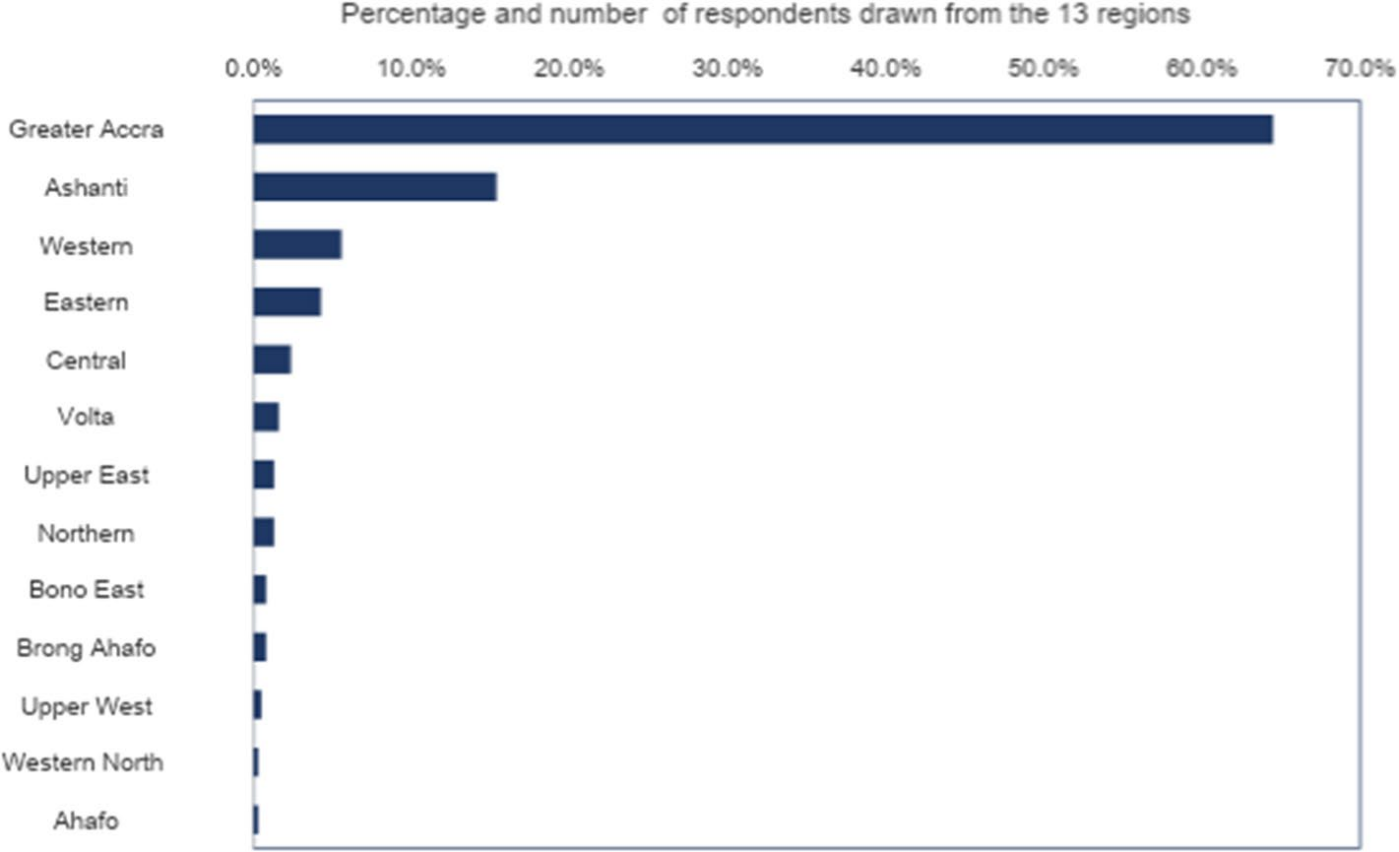
Distribution of respondents across Ghana

An initial review of the 376 responses revealed that 362 representing 96.28% had completed either tertiary or post-tertiary education and only 14 people, representing 3.72% had completed either basic or secondary education. Owing to the disproportionate representation of renters with higher educational attainment, most of whom were graduates, we labelled them as ‘graduate renters’ and based the article on their responses alone. The regional distribution of respondents is provided in Fig. 2.

We analyzed the close-ended questions using simple descriptive statistics, mainly frequencies while the open-ended questions regarding strategies renters have adopted to mobilise funds to pay the AR were analysed using thematic content analysis (Leech & Onwuegbuzie, 2007). This entailed first looking out for the keywords renters used to describe the strategies they used in raising funds to pay the AR and grouping them into common themes following insights from both social capital and rational choice. We then used simple descriptive statistics mainly frequencies and percentages to show the proportion of respondents who chose each strategy. In elaborating on the strategies, we teased out relevant open-ended responses that supported the strategies being reflected upon. All quotes were anonymized and respondents’ gender, age, marital status, type of housing lived in and region of residence were used to provide relevant context for the quotes given the general limited qualitative information respondents provide in surveys.

## Findings

The findings are organized into four parts. The first part presents the demographic characteristics of the study participants. This is followed by findings relating to their housing situation. In part three, we show how participants mobilize funds to pay the advance rent, while part four presents strategies that participants who found the payment of rent advance difficult have instituted to pay the next advance rent without facing difficulties.

### Respondents’ demographic information

The demographic information shows that more than half (57.7%) of the participants identified with the Akan ethnic group, which is consistent with the 52.5% national statistics (Government of Ghana, 2019). Regarding gender, nearly 60% of the participants were males. The mean age of the participants is 32.35 years which is indicative of the youthful population of Ghana and the fact that rental housing generally appeals to young people (Gilbert, 2016; Kendig, 1984). There were more married people (49.2%) than those who had never married (47.2%). The remaining 3.6% were either separated, divorced or widowed. In terms of educational attainment, over half (56.4%) of participants have completed tertiary education while the remaining 43.6% hold a post-tertiary qualification. Nearly 9 in every 10 of the participants were employed. This finding does not necessarily reflect the graduate employment situation in Ghana where most graduates remain unemployed (See Zakaria & Alhassan, 2019). The study found that the majority of respondents (exactly 50%) work in the private formal sector while the public sector (41.2%) and the private informal sector (7.7%) came second and third respectively. We also found that the average household expenditure of the participants was GHȼ1,960.73 (US$345.31), which is 83% higher than the national average household expenditure of GHȼ1,071.42 (US$188.69) (See Government of Ghana, 2019 p.198). This substantial difference may be partly because the study focuses on only graduates instead of the general public.

### Respondents housing circumstance

Regarding respondents’ housing circumstances, the majority of the participants (42%) live in flats or apartments while a total of 32.6% live in compound houses comprising 19.1% chamber and hall and 13.5% single room (see Table 1). These findings differ sharply from recent national housing statistics where the majority of Ghanaians (about 58%) live in compound houses and a little more than 3% live in flats/apartments (Government of Ghana, 2019). This finding also chimes with the recent transformation taking place in the rental housing market where traditional compound houses are being “upgraded” to become flats or apartments (Asante & Ehwi, 2020). Consequently, it is unsurprising that the majority of participants now have exclusive access to a bathroom (92.3%), toilet (90.6%), kitchen (85.1%), electric meter (74.9%), water (64.4%) and a storeroom (31.5%). The average household size of 4.1 is fairly consistent with the national average size of 4.4 (Government of Ghana, 2013). The average number of bedrooms occupied by participants is 2.27 and this command a monthly rent of GHȼ 535.11 (US$94.24). Also, the average period over which participants have to pay the AR is 1.93 years which fairly approximates the 2 years widely reported in the Ghanaian rental housing market (Arku et al., 2012; Ehwi et al., 2020). Consistent with Ehwi et al. (2020)’s finding, participants paid an average AR of GHȼ 12,016.53 (US$ 2116.26).

**Table 1:** Housing information of study participants

Housing information	Frequency (N=362)	Percentage/ standard deviation
*Type of house*		
Flat/apartment Compound house (chamber & hall) Detached house Compound house (Single room) Semi-detached house	152 69 51 49 41	42 19.1 14.1 13.5 11.3
*Exclusive access and use of*		
Bathroom Toilet Kitchen Storeroom Electric meter Water	334 328 308 114 271 233	92.3 90.6 85.1 31.5 74.9 64.4
Average household size	4.10	4.917
Average number of bedrooms	2.27	(2.103)
Average monthly rent (in GHȼ)	535.11	(572.76)
Average rent advance period (in years)	1.93	(1.479)
Average rent advance paid (in GHȼ)	12,016.53	(18,980.21)

*Source*: Authors’ online survey (June 2020)

### Current sources of funds graduate renters use to pay the advance rent

As earlier indicated, previous studies (Arku et al., 2012; Owusu-Ansah et al., 2018) have found that tenants, irrespective of their income level, mostly relied on bonding (help from relatives) and bridging social capital (employees and banks) to pay AR. Contrary to the findings of existing studies, we found that the overwhelming majority of graduate renters in Ghana (81.5%) used their savings to raise funds for all or part payment of their AR. Table 2 shows that this group of renters mobilized an average of 80.14% of their AR from personal savings. Graduate renters in Ghana and SSA more broadly enjoy a favourable social standing than non-graduates. This, we argue, makes it difficult for them to entirely fund their AR with both bonding and bridging capital. Therefore, personal savings is the most reliable and predictable funding alternative that graduate renters draw on to pay AR. This is reflected in the disproportionate average percentage that personal savings contribute towards paying the AR relative to both the bonding and bridging social capital sources (See Table 3). Our findings suggest that the first rational option for renters who are financially resourceful is the use of only personal savings to pay AR. Renters would continue to resort to this funding source in the future if their financial status remains relatively the same.

**Table 2:** Respondents’ sources of funds used to pay advance rent

Rent advance mobilization strategies	Descriptive statistics per strategy				
	N	Min	Max	Mean	SD
Only personal savings	295	10	100	80.14	27.05
Only bonding social capital	77	0	100	47.60	30.70
Only bridging social capital	65	0	100	58.17	27.96
Both personal savings and bonding social capital	321	10	100	77.96	27.49
Both Personal savings and bridging social capital	322	0	100	80.32	26.51

*Source*: Authors’ online survey (June 2020)

Further analysis of the data, as shown in Table 3, revealed that not all graduate renters can raise the full amount of AR from their savings. The second rational option for these graduate renters is to fall on either the bonding or bridging social capital to raise part of their AR. We found that the likelihood or success of graduate renters raising part payment of AR through bonding social capital is relatively the same as bridging social capital. Since most of the sampled graduate renters are employed, they can meet the formal requirements expected by employers and financial institutions, thus making bridging social capital as accessible as other forms of social capital. This demonstrates that, while the extant literature suggests that bonding social capital is often more accessible than bridging social capital, graduate renters in Ghana are indifferent and adopt both in equal measure.

**Table 3:** Breakdown of sources of funds graduate renters use to pay the advance rent

#	Sources of funds	Frequency	Percentage
1	Only personal savings		
	Yes No	295 67	81.5 18.5
2	Only bonding social capital (loans from friends, families and faith-based groups + gift from relatives)		
	Yes No	77 285	21.3 78.7
3	Only bridging social capital (loans from employer + banks/financial institutions		
	Yes No	65 297	18 82
4	Personal savings and bonding social capital		
	Yes No	321 41	88.7 11.3
5	Personal savings and bridging social capital		
	Yes No	322 40	89.0 11.0

Source: Authors’ online survey (June 2020)

We found that some study participants, by being unemployed, newly-employed or a fresh graduate, had very little personal savings to support the payment of AR. Consequently, the rational option for graduate renters (about 21.3%) who found themselves in this situation was to raise on average 50% of their AR solely from their bonding social capital, albeit some successfully mobilized 100% of the AR amount needed from the same source. Similarly, some employed graduate renters, as a result of the lack of planning, a coincidence of AR payment with other pressing needs or some unforeseen circumstance, did not have adequate savings to pay AR. Due to their employment status, this cohort representing about 18% of respondents, mobilized an average of 60% of the AR funds through their bridging social capital.

### Future strategies and sources of funds graduate renters use to pay rent in advance

This section relied on the coded open-ended responses of renters regarding their future strategies to pay the AR. As indicated in Table 4, we found that 174 respondents, representing 48.1% indicated that they faced difficulty raising funds for their AR while the remaining 51.9% said they did not. Among those who had difficulty paying the AR, we enquired about the strategies they will or have initiated to avoid future difficulties in raising funds for the AR payments. Findings revealed that none of the respondents considered using bonding capital as an alternative source of raising AR funds in the future. This, for us, goes to affirm our postulation that graduate renters, having previously used their bonding social capital to access a basic need like housing, find it difficult to fall on them again for the same expenditure. Rather, the strategies identified were oriented towards savings, investments, reducing expenditure on basic needs and bridging social capital.

**Table 4:** Strategies adopted to mobilize funds for future rent advance payment

Strategies adopted for future Rent Advance payment (Respondents who found the payment of RA difficult	Frequency	Percentage (%)
*Only savings*		
Saving with a specific plan	65	37.4
Saving without a specific plan	33	19
Sub-total	99	56.9
*Strategies aside savings and social capital*		
Investing in (high return) short-term assets	14	8.0
Finding a new job or source of income	13	7.4
Reducing expenditure on basic needs	6	3.4
Negotiating for a shorter lease	4	2.3
Moving into a cheaper house	1	− 0.0
Praying to God for help	1	− 0.0
Sub-total	39	22.4
*No Strategy*	21	12.1
*Savings with other strategies (including bridging capital)*		
Saving and building a house for oneself	10	5.7
Saving and borrowing from employers and banks	6	3.4
Sub-total	16	9.1
Total	174	100

*Source*: Authors’ online survey (June 2020)

To start with, we found that relying exclusively on one’s savings was still the predominant future strategy (56.9%) renters are considering mobilizing funds to pay AR. For example, a 29-year old, single female who lived in a flat, working in the financial and insurance industry and residing in Western Region said ‘every month, I save 19% of my salary towards the AR payment’. Also, a 30-year old married woman who lived in a flat/apartment and worked in the real estate sector commented that ‘I save about 50% of my salary every month to cater for the AR’. Further analysis revealed that the savings strategy bifurcates into ‘saving with a specific plan’ (37.4%) and ‘saving without a specific plan’ (19%). The difference between these two forms of savings lies in the respondents clarifying exactly how much will be saved, in terms of amount or percentage of amount over a specified period. Regarding those who saved without a specific plan, this statement by a 26-year old single female from the Volta Region is illustrative: ‘I have started saving some money early and also increased the proportion of monthly saving.’ For those without a specific plan, a 42-year old male who is a quantity surveyor said that ‘I do not have a specific savings plan. When I work for a client and the charge is huge, I save more. But when it is a small job and the charge is small, I save a small proportion.’

The next most popular strategy renters are considering for the payment of AR are those that are not necessarily savings-based. Some (8%) of the respondents indicated that they will invest in short-term assets. A 42-year old engineer who lives in the Ashanti region remarked that ‘I have one more year to the end of my tenancy. In the next two weeks, I will go to the bank to invest some monies in a 91-day treasury bill. I will instruct my bank to roll over the investment till I need it to pay my AR.’ While some were considering low risk and low return government instruments, others were looking for high risk and high return short-term investments. A 38-year old businessman commented that ‘A friend recently told me about a new company in Ghana. They are into farming. You invest any amount of your choice in the company and every month they pay you 50% of your investment. I have arranged with my friend to visit the company and when we go there I will invest some monies.’ Careful consideration of this strategy would reveal that they are underlined with several uncertainties and risks which in our view, may outweigh those found in social capital. For example, events heralding the recent financial sector clean-up in Ghana, notably the infamous ‘Menzgold’ saga (Yalley, 2021), makes strategies such as investing in high return short-term assets risky.

More so, some respondents (5.7%) were of the view that some funds could be raised for AR by reducing expenditure on basic needs or moving into cheaper accommodation. Regarding the former, a 34-year old female teacher said that ‘I spend too much on changing my clothing every month. I have decided to cut back on these monthly expenses to save money for the payment of my next advance rent’. Concerning the latter, ‘I’ll move to a cheaper place’, says a 24-year old single woman living in a compound house in Greater Accra and working in the financial and insurance sector. It is believed that by moving into cheaper accommodation, the quantum of money to be raised for the payment of AR will be reduced. For some respondents (2.3%), negotiating for a shorter lease is a good strategy to reducing the burden of AR going forward. A 48-year old journalist in Accra indicated that ‘I currently pay 2 years advance rent for my accommodation. I have started engaging my landlord about the possibility of paying the rent every 6 months. He has promised to give me feedback next month.’

Rather surprisingly, 12.1% of the renters had no strategy in the sense that they merely echoed sentiments of being either fed-up or not knowing what to do potentially because of the difficulty in finding well-paid jobs as well as peculiar household and family circumstances. This insight emerged from a comment by a 31-year land surveyor: ‘*I have no strategy due to several factors. My current job does not pay well. The salary is not enough to take care of myself, my wife and our 4 kids. My 85-year mum is sick most of the time and I am responsible for her medical bills and upkeep. How can I save under this circumstance? For the past 3 years, I have applied for several jobs to improve my income but all to no avail’*. Another 29-year old married woman living in a detached house in the Volta Region also observed that ‘*I haven’t found any (strategy) because now I am both unemployed and still searching for jobs’*. Predominantly, these cohorts echoed sentiments of being entrapped, helpless and feeling the only way out is to seek divine intervention. The married woman respondent said that ‘we are praying to God for help’.

Although bonding social capital was not an option for funding AR payment in the future, some graduate renters (9.1%) indicated that bridging social capital will be an important source of funding to supplement their savings. Emphasis was put on loans from employers and banks. Two respondents remarked that:

I will save more than I previously did and if possible or necessary, access a loan from my bank

‘My strategy entails saving more than I previously saved and also apply for a loan from my employer before the advance rent is due’.

Due to the burden of regularly raising funds for AR, some respondents (5.7%) believe that funds should not only be raised to pay AR but also to build a house to exit the rental market. According to a 27-year old married man living in a flat in the Ashanti Region: ‘What I have been doing for some time now is channel some of my savings and other funds into building a house. I have done this for the past 5 years so very soon I will move to my own house and stop paying advance rent.’ Consequently, the payment of AR should not be a perpetual activity but should end at a point when the renter completes the construction of his house.

## Discussion

Our study has uncovered several findings which depart from received wisdom in Ghana’s rental housing market and it is important to reflect on why this seems to be the case and the theoretical contributions it makes. First, we must concede that graduate renters are not representative of the entire rental household population in Ghana and hence our findings are not generalisable beyond the sample. Rather, it applies to a sub-sector of the rental housing market that has not been previously explored as existing housing studies have tended to draw on factors such as residential neighbourhood classification (Addo, 2016; Gavu & Owusu-Ansah, 2019), whether the rental units comply with planning permission (Arku et al., 2016) or the houses were built by petty landlords, the state, estate developers or employers (Adu-Gyamfi et al., 2019) to classify tenants. These different approaches to classifying renters yield unique characteristics which have implications for the findings reported.

For example, while the official figures indicate that compound houses are the predominant accommodation type in Ghana, accounting for over 50% of the 3.4 million stock of housing (Government of Ghana, 2019), our findings revealed that it was rather flats and apartments that seem popular among graduate renters. This shows a changing housing preference within different sub-sectors of the rental housing market partly because of the litany of problems that characterize living in compound houses such as queuing to use toilets and baths during the rush hours of the day (See Danso-Wiredu & Poku, 2020; Konadu-Agyemang, 2001) and petty squabbles when allocating utility bills (Asante & Ehwi, 2020).

In line with the above, we should also expect the sources of funds and strategies for mobilising funds by graduate renters to differ from the general renter population in Ghana. Indeed, by drawing conceptual insights from both rational choice and social capital theory, we have shown that temporal analysis of the sources of funding drawn upon to pay the AR is important as previous studies (Arku et al., 2012; Owusu-Ansah et al., 2018) have overlooked this insight, leading to claims that social capital including bonding and bridging capital is the most preeminent source of funding renters draw upon to pay the AR. We have demonstrated that it is rather savings that is the most preferred source of funding for paying the AR both currently and in the future. This departure, we argue, is strongly linked to this novel graduate-dominated sub-market of the rental housing market we focus on in this article. Indeed, with 46.3% of our sample having attained post-tertiary educational qualifications (Masters and PhDs), one can appreciate why it will be challenging for this new cohort to rely mostly on bonding and bridging social capital to pay their AR. This is not to suggest that no graduate renter uses social capital to help them pay their AR; some still do. However, within the Ghanaian context, research has shown that attaining higher education is one of the guaranteed ways people can join the middle class (Budniok & Noll, 2018). Thus, the general expectation for graduates to lead lifestyles reflecting a certain degree of comfort and independence, makes it difficult for them to rely mostly on social capital as a source of income to fund the AR payment and not from one’s savings.

The above insight brings out a revealing theoretical contribution in the following manner. First, it shows how the assumptions underpinning rational choice decision making, including the fact that people’s actions are primarily motivated by self-interest, opportunism and calculative actions can be significantly circumscribed by social capital in some circumstances. Due to a desire to protect their image as highly-regarded members of the Ghanaian society (the emerging middle-class), graduate renters would often shy away from making it a practice to always use their social capital to meet their AR payment. Indeed, while rational choice thinking would suggest that opportunistic tendencies and desire to avoid hardship would incentivise graduate renters to fall on their social capital to pay the AR, they seem to have realised that this practice risks damaging their image and the social capital they fall on. This is because other contributors of the social capital that they (graduate renters) draw upon could easily become fed up with their lack of personal responsibility for their financial planning and hence devise means to dissolve the social capital. Thus, to avoid losing both their hard-earned reputation and social capital, graduate renters, as our study shows, would instead rely heavily on their savings both currently and as a future source of income, and only draw on their social capital when needed.

This puts graduate renters between a rock and a hard place in that, despite the reputation they command, their economic circumstances are often not commensurate, leading to the financial precarity that some of the graduate renters in this study have echoed. From the foregoing, it would seem appropriate that the findings offer an opportunity for both policy-makers to pay attention to the pay structure and salaries of graduates in the Ghanaian economy as it appears there is a misalignment between societal expectations of graduates and what their economic circumstances will reasonably allow. Indeed, the salaries of graduates at both entry and experienced levels deserve urgent attention by policymakers to align it with the current macro-economic indicators in the country, chiefly inflation. We contend that the current calls by the University Teachers Association of Ghana (UTAG) (JoyNews, 2021) and other professional bodies present an opportune time for this long-overdue conversation to be had.

Also, to us, the observation that some graduate renters are not considering using social capital but rather their savings and other strategies not related to social capital to pay their future AR could portend some undesirable outcomes, such as poor household wellbeing following some graduate renters cutting down on basic consumption, buying litigation instead of land to start a building project (due to multiple sales of the same piece of land), losing hard-earned income to fraudulent high-return short term investment portfolios.

## Concluding remarks

Following the limited empirical attention given to the strategies renters in Ghana adopt to mobilise funds to pay their AR, the study sought to explore the sources of funds graduate renters draw upon to pay their AR. We also explored how their sources of funds and strategy for mobilising funds change over time. We found that, contrary to the popular claim that social capital in the form of both bonding capital (financial help from relatives and friends) and bridging capital (loans from employers and banks) remain the most preferred source of funds and strategy for paying the AR, this study reveals that for graduate renters, it is rather savings that they mostly draw upon to pay their AR both currently and as a future source of income. We found that while some graduate renters used their social capital exclusively or in combination with other strategies to obtain funds to pay their AR, it never overshadows savings. We interpret this new finding as being driven by graduate renters’ need to protect both their social reputation and also the social capital they draw upon, rather than using them opportunistically as rational choice theory seems to suggest. We have also highlighted in our analysis that this new approach to funding the payment of the AR has portended precarity for some graduate renters who have to make important trade-offs on necessities such as cutting down consumption to save more to pay the AR.

We contend that our findings and the threats of demonstrations by professional groups like UTAG open the door for the government to urgently consider the salary situation of graduates in Ghana as their enviable social reputation does not reflect in their economic circumstances, despite the expectation thrust upon them. We have also made a theoretical contribution by showing how axioms of rational choice decision-making can be circumscribed by the graduate renter’s image and the fear of damaging their social capital. Also, we have drawn attention to the need to view social capital, not as a static concept but one that has temporal dimension for different social groups. More empirical studies are warranted to fully understand why graduate renters fail to use their bonding social capital in the future, although the alternatives they adopt seem less reliable and risky.

It is further worth highlighting that this study’s findings contribute to the emerging body of empirical scholarship calling for studies into sub-markets within the rental housing market [See for example, Ehwi et al. (2020) on the need to differentiate between first-time and regular renters, Gavu & Owusu-Ansah (2019) for different housing features in different residential classifications in Accra-Ghana and Asante & Ehwi (2020) for understanding ongoing changes in the amenities supplied, the rent charged, tenant selection and use of shared spaces between traditional and transformed compound houses in Bantama, Kumasi]. This emerging scholarship is important because it provides novel insights that challenge popular notions regarding housing markets in Ghana, including but not limited to the popular type of housing in Ghana, the employment of sectors of renters, their housing characteristics, among others. While national census data are useful for painting an overall picture of the housing market in Ghana, we contend that smaller sample studies focusing on specific sub-markets of rental housing market in Ghana can bring to the fore insights previously unknown or overlooked in the extant literature.

In closing, it is worth highlighting some methodological limitations of the study. Firstly, using an online survey for data collection meant that the study could not have captured every renter except those who fell within the author’s social networks and those who were snowballed from the social networks. While this limits our ability to generalize our findings as being representative of the entire rental households in Ghana, the online survey made it possible for us to identify this novel sub-market previously unknown and which using traditional in-person data collection might not have been uncovered.
